# Impact of high intensity interval exercise with and without heat stress on cardiovascular and aerobic performance: a pilot study

**DOI:** 10.1186/s13102-023-00682-8

**Published:** 2023-07-11

**Authors:** Alexs A. Matias, Isabelle F. Albin, Leah Glickman, Peter A. Califano, Justin M. Faller, Gwenael Layec, Stephen J. Ives

**Affiliations:** 1 Department of Health and Human Physiological Sciences, Skidmore College, 815 N. Broadway, Saratoga Springs, NY 12866 USA; 2 Department of Kinesiology, University of Massachusetts at Amherst, Amherst, MA USA; 3 Institute for Applied Life Sciences, University of Massachusetts, Amherst, MA USA

**Keywords:** Blood pressure, Running, Vascular stiffness, Pulse wave velocity, Heat acclimation

## Abstract

**Background:**

Heat stress during aerobic exercise training may offer an additional stimulus to improve cardiovascular function and performance in a cool-temperate environment. However, there is a paucity of information on the additive effects of high-intensity interval exercise (HIIE) and acute heat stress. We aimed to determine the effects of HIIE in combination with acute heat stress on cardiovascular function and exercise performance.

**Methods:**

Twelve active (peak O_2_ consumption [VO_2peak_]: 47 ± 8 ml·O_2_/min/kg) young adults were counterbalanced to six sessions of HIIE in hot (HIIE-H, 30 ± 1 °C, 50 ± 5% relative humidity [RH]) or temperate conditions (HIIE-T, 20 ± 2 °C, 15 ± 10% RH). Resting heart rate (HR), HR variability (HRV), central (cBP) and peripheral blood pressure (pBP), peripheral mean arterial pressure (pMAP), pulse wave velocity (PWV), VO_2peak_, and 5-km treadmill time-trial were measured pre- and post-training.

**Results:**

Resting HR and HRV were not significantly different between groups. However, expressed as percent change from baseline, cSBP (HIIE-T: + 0.9 ± 3.6 and HIIE-H: -6.6 ± 3.0%, *p* = 0.03) and pSBP (HIIE-T: -2.0 ± 4.6 and HIIE-H: -8.4 ± 4.7%, *p* = 0.04) were lower in the heat group. Post-training PWV was also significantly lower in the heat group (HIIE-T: + 0.4% and HIIE-H: -6.3%, *p* = 0.03). Time-trial performance improved with training when data from both groups were pooled, and estimated VO_2peak_ was not significantly different between groups (HIIE-T: 0.7% and HIIE-H: 6.0%, *p* = 0.10, Cohen’s d = 1.4).

**Conclusions:**

The addition of acute heat stress to HIIE elicited additive adaptations in only cardiovascular function compared to HIIE alone in active young adults in temperate conditions, thus providing evidence for its effectiveness as a strategy to amplify exercise-induced cardiovascular adaptations.

## Background

Acute exposure to high ambient temperatures (e.g., hot-humid conditions) can overwhelm heat dissipating and regulatory mechanisms in the body, thereby inducing thermal load known as *‘heat stress’*. The ensuing physiological strain, notably, impaired cardiovascular (CV) [1] and metabolic function [2], is associated with acute reductions in functional performance in both hot [3] and temperate [4] conditions. At its extreme, heat strain can significantly increase heat-related illness morbidity and mortality risk [5]. However, controlled, and repeated exposure (> 5 days) to whole-body hyperthermia has been shown to induce physiological adaptations, e.g., increased sweat rate, that mitigate the deleterious effects of exercise in the heat [6]. Heat acclimation (HA) may even promote cross-adaptation in temperate conditions [7] and a greater resistance to various exercise stressors (e.g., hypoxia) [8, 9].

Over the past decade, the additive effect of combined exercise and heat has emerged as a novel and attractive strategy to improve endurance performance not only in hot conditions, but also in temperate conditions [10–14]. Indeed, reductions in basal core temperature (T_core_) [10, 15, 16], a greater sweat rate [7, 10] and cutaneous blood flow [7], which collectively augment cutaneous heat loss, are hallmark thermoregulatory responses to exercise HA. These adaptations translate to improvements in cardiac filling pressures and blood velocities [17], plasmatic volume [11, 16, 17], heart rate (HR), and blood pressure (BP) [18]. Reductions in arterial stiffness and improved endothelial function also follow passive chronic heat exposure [19, 20], suggesting improvement in CV risk profile [21] in sedentary adults. Taken together, these findings suggest that combined exercise and HA may influence several factors determining O_2_ supply to the active muscles (e.g., cardiac output, vascular resistance) and blood flow redistribution, which could theoretically amplify the adaptations induced by exercise training alone.

To this regard, a seminal study by Lorenzo et al. demonstrated that 10 sessions of low- to moderate-intensity cycling (elite endurance cyclists; maximal oxygen consumption [VO_2max_]: ~ 67 ml/kg/min) in the heat (38˚C) induced plasma volume (PV) expansion, augmenting stroke volume (SV) and ventricular compliance, which in turn improved cardiac output (CO) and VO_2max_ in temperate conditions [11]. However, this effect was subsequently attributed to the greater relative intensity of the training modality in the heat rather than an additive ergogenic benefits from heat stress. In fact, using a counter-balanced crossover study design with exercise intensity matched between conditions, VO_2max_ and time-trial performance were unchanged after 10 consecutive sessions of moderate-intensity continuous cycling in the heat (38˚C) in trained individuals (VO_2max_: ~ 61 ml/kg/min) [22]. Importantly, the thermophysiological adaptations to exercise coupled with thermal load have primarily been investigated using low-intensity, long term (> 10 days) HA (LTHA) protocols, which are time intensive and difficult to implement [23].

In this regard, high-intensity interval exercise, i.e., HIIT, is an effective, time-efficient training paradigm [24]. HIIT incorporates intervals of quick, high intensity exercise bouts, i.e., exercise above the lactate threshold, and long, lower intensity *“active recovery”* exercise bouts repeated in succession. Conventional HIIT sessions last no more than 30 min and has been known to elicit robust metabolic (e.g., improved fat oxidation) [25, 26] and cardiovascular (e.g., improved blood pressure regulation and vascular function) [24, 27] adaptations. Interestingly, whether the physiological adaptations induced by short-term HA and high-intensity exercise training can translate into improved performance in temperate conditions or elicit antagonistic effects is currently still debated [28, 29].

For example, using a parallel study design, Karlsen et al. reported no significant effects of 2 weeks of HA (34˚C) on VO_2max_ and a 43 km cycling performance test in temperate conditions outdoor in competitive cyclist (VO_2max_: ~ 4.8 l/min) training ~ 15 h. a week, including 2.5 h. of high-intensity interval exercise [30]. Although this field-based study provided important insight into HA in well-trained athletes, the interaction between high-intensity exercise training and HA was not directly tested as the participants maintained their training routine during the study period. Using a design to test the interaction between HA and 3 weeks of high-intensity training (3 sessions per week, ~ 33˚C), McCleave et al. documented a 3.3% improvement in the performance during a 3 km running test conducted in temperate condition outdoor in well-trained runners (peak oxygen consumption [VO_2peak_]: ~ 62–65 ml/kg/min) [31]. Surprisingly, running performance was not improved immediately after the intervention, but 3 weeks later, i.e., when plasma and blood volume values had returned to baseline, such that the physiological adaptations responsible for this delayed performance improvement were unclear.

To date, the effects of combined high-intensity interval exercise (HIIE) and short-term HA on performance, and the underlying physiological adaptation, in a temperate environment are still poorly understood. Furthermore, studies have primarily focused on elite/semi-elite athletes, although recreational athletes routinely exercise in hot environments. Accordingly, the goal of the present pilot study was to determine the potential interaction of low-volume HIIE and short-term heat exposure on CV function (i.e., heart rate [HR], HR variability [HRV], blood pressure [BP], peripheral mean arterial pressure [pMAP]), arterial stiffness (pulse wave velocity [PWV]), whole-body VO_2peak_, and performance during a 5 km treadmill time-trial in active young adults. Based upon prior findings [7, 11, 31], we hypothesized that HIIE combined with heat exposure would improve, to a greater extent, CV function and aerobic performance compared to a temperate group undergoing a HIIE program at the same relative intensity.

## Methods

### Participants and general procedures

Twelve physically active young adults (males = 6, females = 6) were recruited for this study. Exclusion from participation included current or recent (< 6 months) smokers, and those with any past or present history of cardiovascular diseases and/or heat-related contraindications. Health history was assessed using self-administered questionnaires (American College of Sports Medicine [ACSM] Pre-Participation Screening, Physical Activity Readiness Questionnaire [PAR-Q], Heat Tolerance Questionnaire). Physical activity was defined as regularly engaging in a minimum of 30 min of moderate intensity aerobic activity, preferably running, at least 3 days/week, in accordance with ACSM guideline. All participants provided written informed consent prior to participation. Approval for this study was granted by the Human Subjects Institutional Review Board of Skidmore College (#1712–675) and was conducted in accordance with the most recent revisions of the Declaration of Helsinki.

**Fig. 1:**
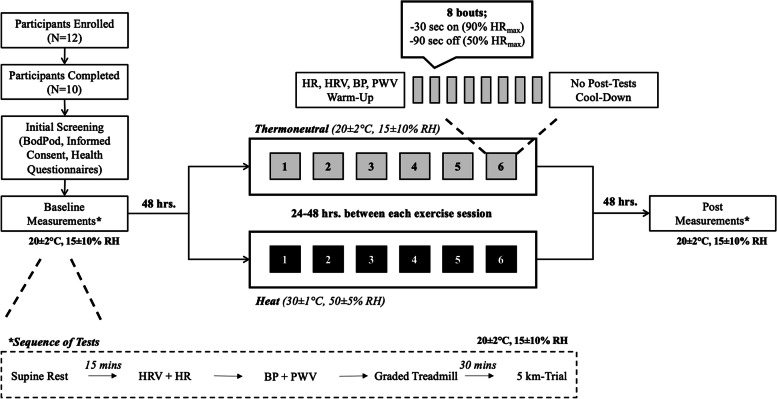
Schematic of the experimental design, detailing the different visits, timing between visits, and tests being conducted (and the order of tests)

To characterize participants, height and weight were measured using standard techniques, and body fat percentage, fat mass, and fat free mass were measured using air displacement plethysmography (BodPod, CosMed, Chicago, IL.) with documented reliability [32]. Participants were asked to refrain from consuming alcohol, caffeine, or other ergogenic supplements, and from engaging in strenuous exercise, at least 12 and 24 h. prior to each testing session, respectively. All trials were performed at least 2 h. post-prandial, and participants were asked to arrive well hydrated. To ensure hydration status, participants were instructed to consume at least 30 ml/kg/day of water 24 h. prior to each testing session, which has been confirmed before with measures of urine specific gravity (USG) [33]. To allow for adequate recovery, a minimum of 48 h. was allocated between all visits, but the whole study confined to three weeks (see Fig. 1. for a detailed description of the order of experimental tests and timing). Within subjects, all tests and exercise sessions were completed during the same time of day to minimize the impact of diurnal variations, under identical experimental conditions (e.g., normobaric [~ 760 mmHg]), with the only difference being ambient temperature. Training during lead-in phase of the study was not controlled for; however, no difference in VO_2peak_ was observed at baseline, and participants reported similar prior exposure to exercise-heat stress, suggesting comparable physical fitness levels.

### Study design

The current study was conducted using a counterbalanced, parallel, between-subjects design (Fig. 1.) Participants were randomly assigned into two separate groups performing six sessions of HIIE in either 1) hot and humid conditions (HIIE-H: 30 ± 1 °C, 50 ± 5% RH, Wet Bulb: ~ 22 °C) or 2) a controlled, temperate environment (HIIE-T: 20 ± 2 °C, 15 ± 10% RH, Wet Bulb: ~ 8 °C). The temperature and RH combination for both the HIIT-H and HIIT-T groups were chosen to match the wet bulb temperatures reported in the literature [11, 22], which has been shown to induce significant HA. Six exercise-heat sessions were chosen based on evidence suggesting that the majority of thermophysiological adaptations [12, 34] are induced by six days of HA, with some performance benefits [10, 13] also observed at this duration. Exercise sessions performed under heat stress were conducted in a calibrated climatically controlled environmental chamber (Darwin Chamber, Saint Louis, MO). All other pre- and post-training assessments were performed in a temperature regulated laboratory, under the same conditions as HIIE-T (Fig. 1.). All testing and training sessions were conducted between late winter-early spring (average temperature =  ~ 1 °C), thus reducing any potential confound of partial HA from ambient environmental exposure.

### Graded exercise protocol

To determine HR zones for training, and to estimate peak aerobic capacity (VO_2peak_), participants completed a graded treadmill (Trackmaster) exercise test before and at the completion of the training intervention. Briefly, upon arrival to the laboratory, participants were equipped with a HR monitor (H7, Polar USA, Lake Success, NY) and oscillometric Mobil-O-Graph pulse wave analysis system (IemGmbH, Stolberg, Germany), and rested in a seated position for 15 min (ambient conditions: 20 ± 2 °C, 15 ± 10% RH) while baseline cardiovascular (HR, HRV, BP, and PWV) and perceptual measurements (rate of perceived exertion [RPE], thermal sensation [TS]) were recorded. Participants were then equipped with a mouthpiece connected to a metabolic cart spirometry and gas analysis system for assessment of indirect calorimetry (TrueOne2400 Metabolic Measurement System, ParvoMedics, Sandy, UT), a system with documented reliability and validity [35]. The exercise protocol started at an initial speed of 5.5 mph, increasing by 0.5 mph every two minutes. The incline of the treadmill was initially kept at 0%, and adjusted for comfort to no more than 2%, throughout the entire test. Due to institutional safety constraints, testing ceased once participants reached 90% of their estimated age-predicted HR_max_, which was calculated according to the equation proposed by Fox et al., (1971) (HR_max_ = 220 – age) [36] and has been validated in young adults. As a result, not all participants reached a VO_2_ plateau, and thus data is presented as VO_2peak_. Obtaining HR across a range of speeds allowed direct determination of exercise intensities for training sessions using the linear relationship between HR and intensity (i.e., running speed).

### Assessment of exercise performance in a temperate environment

Participants rested for ~ 30 min and consumed water ad libitum following the graded exercise test, after which aerobic endurance performance was assessed by a 5-km running time-trial test on a motorized treadmill (ambient conditions: 20 ± 2 °C, 15 ± 10% RH). This exercise modality and distance was chosen for reliably monitoring aerobic running performance in trained athletes [37]. Participants first completed a five-minute warm up at their self-selected pace and then immediately began the time-trial test. Speed, duration, and distance were visible to participants at all times, and intensity self-determined at a fixed rate for the entire test. Equal verbal encouragement was provided to all participants. To quantify meaningful changes in aerobic endurance performance and perceptual markers, time, speed, TS, RPE, and HR were recorded every 30 s.

### HIIE protocol

Participants reported to lab and were equipped with an HR monitor to assess basal HR and HRV. For both groups, this’baseline’ assessment of CV and cardiac autonomic function, respectively, was conducted after a quiet, seated rest for 15 min in temperate ambient conditions (20 ± 2 °C, 15 ± 10% RH). Participants then began a light five-minute treadmill warm-up at 3.5 mph, 0% grade, in their respective ambient conditions, immediately followed by HIIE. The HIIE program consisted of eight consecutive bouts of 30:90 intervals; 30 s of high-intensity running (90% of age-predicted HR_max_) followed by 90 s of low-intensity active recovery (50% of age-predicted HR_max_) [38]. HR values obtained across a broad range of speeds during the graded treadmill test were used to characterize individual exercise intensity values, however, speed was adjusted real-time during all exercise sessions to ensure participants exercised at 90% age predicted HR_max_ such that relative intensity was similar between groups. To standardize intensities and monitor health, HR, speed, and grade were recorded every 30 s in real time using portable HR monitors (H7) [39] and the Polar Team application (Polar USA, Lake Success, NY) displayed on a mobile device (iPhone, Apple Inc., CA). This workload-based exercise HA protocol was used due to its effectiveness in inducing favorable physiological and performance adaptations in prior animal [13] and human studies [10, 16, 40].

### Cardiac autonomic function

HRV was used as an estimate of autonomic balance of the heart (i.e., sympathetic and parasympathetic tone) [41] and was collected following a 15-min resting baseline pre- and post-intervention, as well as before each exercise session in temperate conditions (20 ± 2 °C, 15 ± 10% RH). Time domain-based metrics of HRV (root mean square of the successive difference [RMSSD] and standard deviation of the N–N intervals [SDNN]), in conjunction with HR, were collected during five minutes of quiet rest in the supine position, standardized to eight breaths/min (paced breathing via visual feedback from commercially available software [Elite HRV]), using portable HR monitors (Polar H7, Polar Electro), which have been validated against ECG, especially at rest [42]. RMSSD captures the beat-to-beat variance in resting HR, providing a time-domain surrogate of vagal tone (i.e., parasympathetic nervous system function) [43]. SDNN is a composite of variations in both sympathetic and parasympathetic signals. Given limitations of the available software, frequency-domain analysis was not performed. Data were acquired and analyzed using Elite HRV, which has previously been cross-validated with ECG-derived gold standard during supine rest and an orthostatic challenge [44].

### Arterial stiffness and central blood pressure

Assessments of central BP and arterial stiffness (i.e., PWV) were immediately conducted following HRV testing. Using standard techniques, a BP cuff was positioned proximal to the antecubital fossa of the supported arm. Following five minutes of quite supine rest, aortic pulse wave velocity (PWV) and BP (central and peripheral) were recorded and analyzed using the reliable [45] and validated oscillometric Mobil-O-Graph pulse wave analysis system (IemGmbH, Stolberg, Germany) [46]. Central BP is an index of the aortic pressure waveform derived from the peripheral pressure waveform at the peripheral brachial artery using a general transfer function. Assessment of central blood pressures has been suggested to be of greater importance as they more accurately reflect the afterload on the heart and are more closely associated with left ventricular hypertrophy [47]. PWV is considered a key prognostic indicator of CV health beyond traditional risk factors [21].

### Data analysis

Statistical comparisons were performed with commercially available software (GraphPad Prism version 9.1.1, GraphPad Software, San Diego, California USA). Given time constraints and the complexity of an intervention design, 12 participants were recruited, but only 10 (*n* = 5 per group) completed the study. However, a power analyses based on the study by Brunt et al. [20] and using the reported change in MAP pre (83 ± 1 mmHg) vs. post (78 ± 2) heat stress in the present study suggests a *n* = 3 per group (Cohen’s d = 3.16, alpha 0.05, power at min 0.8). Further, the COVID-19 pandemic and associated institutional precautions regarding human subjects research has made additional data collection not possible. Therefore, to account for a low sample size and non-normally distributed data, non-parametric tests were performed where appropriate. Based upon prior evidence indicating a large beneficial effect of combined heat stress and exercise training [7, 11], one-tailed Mann–Whitney U tests were conducted to identify potential group differences in relative changes from baseline. Repeated Measures two x two analysis of variance (ANOVA) was used to determine if main effects were found in group (temperature: heat vs. temperate), time (training: pre vs. post), and any potential group by time interactions on any of the measured variables. For t-test type model, conventions of magnitude of the effect size are 0.2, 0.5, and 0.8 for small, moderate, and strong effect, respectively, for Cohens *d* = (M_2_ – M1)/SD_pooled_. The reported effect sizes also provide context for future research. Alpha was set, a priori, at 0.05 for all comparisons. All data are presented as mean ± standard deviation in the manuscript and tables, and mean ± standard error of the mean (SEM) in figures, unless stated otherwise.

## Results

### Participant characteristics

Participant characteristics are presented in Table 1. A total of 12 participants were initially recruited, but due to timing issues and an injury (outside of laboratory), data presented are for 10 participants (*n* = 5 per group, 3 females and 2 males in each group). There were no significant between-group differences in any anthropometric measures (all, *p* > 0.05, Table 1). Furthermore, no baseline between-group differences were detected for all CV and performance markers (all, *p* > 0.05). In conjunction with a matched training stimulus (i.e., both groups performed HIIE) and similar baseline VO_2peak_ (Fig. 6A), this suggests that any observed changes in CV and performance markers were likely due to heat stress, rather than a baseline difference.

**Table 1:** Participant characteristics

Characteristics	All (*N* = 10)	All (*N* = 10)	Heat (*n* = 5)	Temperate (*n* = 5)	*p*-value
**Sex (F/M)**	**6/4**	**Range**	**3/2**	**3/2**	
Age (yrs.)	22 ± 1	19–30	21 ± 1	22 ± 2	0.33
Weight (kg)	61.2 ± 2.5	47.0–73.0	61.2 ± 0.4	61.3 ± 2.6	0.41
Height (cm)	167.0 ± 3.5	155.4–188.0	166.4 ± 0.4	167.8 ± 3.7	0.40
Fat Mass (%)	15.7 ± 2.4	6.4–21.5	15.1 ± 0.4	16.5 ± 2.7	0.35
Fat Free Mass (%)	51.6 ± 2.8	41.5–93.6	89.9 ± 0.4	83.4 ± 2.6	0.35
BMI (kg·m^−2^)	21.7 ± 0.6	19.5–22.9	21.7 ± 0.4	21.8 ± 0.4	0.36
VO_2peak_ (ml/kg/min)	47.1 ± 8.4	38.0–60.1	47.3 ± 8.7	46.9 ± 9.0	0.48

Data presented mean ± SD

*BMI* Body Mass Index, ***VO***_*2peak*_ Peak Oxygen Consumption

### Heart rate and heart rate variability

There was no significant (all *p* > 0.05) temperature x time interaction for all metrics of HR and HRV (Table 2). Also, there was no main effect of temperature or time for all metrics of HR and HRV (all *p* > 0.05). As expected, there was a similar yet marginal reduction in HR post-training in both HIIE-T and HIIE-H, although this did not reach significance (all *p* > 0.39, Cohen’s d < 0.21).

**Table 2:** Indices of Heart Rate (HR) and Heart Rate Variability (HRV)

	**Temperate (F = 3, M = 2)**		**Heat (F = 3, M = 2)**	
**Variable**	**Baseline**	**Post-Training**	**Baseline**	**Post-Training**
HR (bpm)	74.5 ± 6.1	72.8 ± 6.2	74.6 ± 11.1	72.6 ± 8.5
RMSSD (ms)	51.1 ± 21.4	52.0 ± 25.5	71.6 ± 23.9	65.8 ± 12.5
LnRMSSD (a.u.)	3.9 ± 0.4	3.8 ± 0.6	4.2 ± 0.4	4.1 ± 0.2
SDNN (ms)	68.3 ± 25.4	67.3 ± 24.9	87.3 ± 17.9	97.7 ± 26.9

Data presented mean ± SD

*HR* Heart Rate, *RMSSD* Root Mean Square of the Successive Differences, *LnRMSSD* Natural Log of RMSSD, *SDNN* Standard Deviation of the NN intervals, ***F*** Females, *M Males*

### Blood pressure

**Fig. 2:**
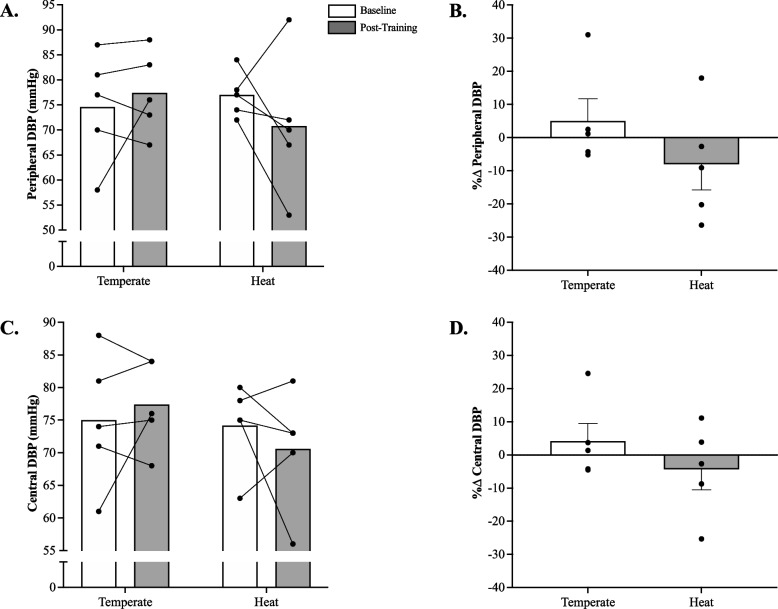
**A** Peripheral Diastolic Blood Pressure (pDBP; Cohen’s d = 0.7) and (**C**) Central Diastolic Blood Pressure (cDBP; Cohen’s d = 0.5) at baseline and post-HIIE training with or without heat acclimation; **B** percent change in pDBP (Cohen’s d = 0.9) and (**D**) cDBP (Cohen’s d = 0.7). HIIE-T (*n* = 5; females = 3, males = 2) and HIIE-H (*n* = 5; females = 3, males = 2). Data presented mean ± SEM

There was no significant (*p* > 0.05) temperature x time interaction for peripheral (*p* = 0.24, η^2^ = 0.05) and central (*p* = 0.32, η^2^ = 0.03) diastolic BP (Fig. 2A,C). Also, there was no significant main effect of temperature or time for peripheral (time: *p* = 0.65, η^2^ = 0.008; temperature: *p* = 0.71, η^2^ = 0.01). and central (time: *p* = 0.84, η^2^ = 0.001; temperature: *p* = 0.42, η^2^ = 0.06) diastolic BP. Likewise, no significant between-group difference in peripheral (*p* = 0.11, Cohen’s d = 0.91) and central diastolic (*p* = 0.35, Cohen’s d = 0.74) BP was documented when expressed as a percent change from baseline (Fig. 2B,D).

**Fig. 3:**
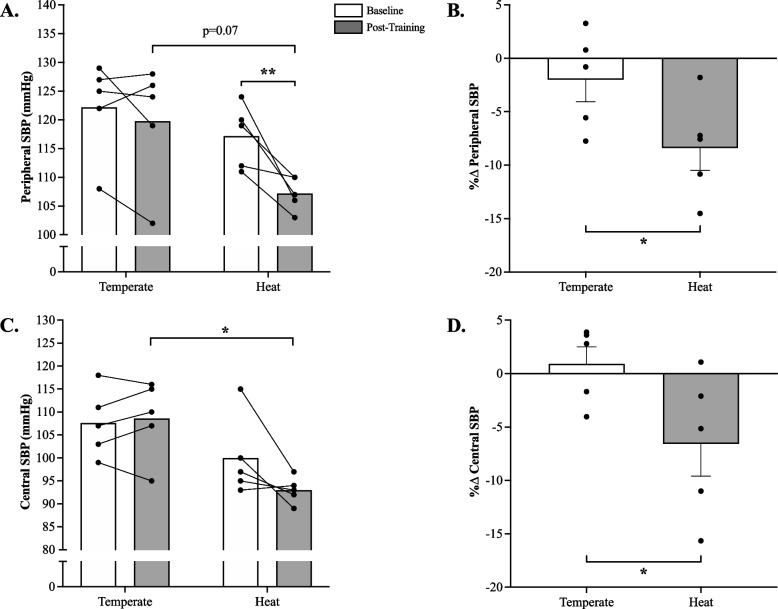
**A** Peripheral Systolic Blood Pressure (pSBP; Cohen’s d = 2.5) and (**C**) Central Systolic Blood Pressure (cSBP; Cohen’s d = 1.2) at baseline and post-HIIE training with or without heat acclimation; **B** percent change in pSBP (Cohen’s d = 1.5) and (**D**) cSBP (Cohen’s d = 1.6). HIIE-T (*n* = 5; females = 3, males = 2) and HIIE-H (*n* = 5; females = 3, males = 2). *p < 0.05. **p < 0.001. A one-tailed Mann–Whitney U Test was used to identify group differences in relative changes from baseline (panel **B** and **D**). Repeated measures 2 × 2 ANOVA was used to identify any potential main effects and interactions (panel **A** and **C**). Data presented mean ± SEM

There was no significant temperature x time interaction for peripheral (*p* = 0.07, η^2^ = 0.47) and central (*p* = 0.07, η^2^ = 0.49) systolic BP (Fig. 3). However, a significant main effect of temperature was detected for central systolic BP only (*p* = 0.02, η^2^ = 0.41), although there was a trend for peripheral systolic BP (*p* = 0.07, η^2^ = 0.25). Central (Fig. 3C; *p* = 0.02, Cohen’s d = 2.8) systolic BP was significantly lower in HIIE-H than HIIE-T post-training, while peripheral systolic BP tended to follow a similar reduction in the HIIE-H group (Fig. 3A; *p* = 0.07, Cohen’s d = 1.8). A significant main effect of time was observed for peripheral systolic BP (*p* < 0.001, η^2^ = 0.13), such that peripheral systolic BP tended to decrease post-training in HIIE-H only (Fig. 3A; Cohen’s d = 2.5). Lastly, non-parametric t-tests revealed that both central (Fig. 3B; *p* = 0.03, Cohen’s d = 1.5) and peripheral systolic BP (Fig. 3D; *p* = 0.04, Cohen’s d = 1.5), expressed as percent change from baseline were significantly reduced (negative percent change) in HIIE-H as compared to HIIE-T.

**Fig. 4:**
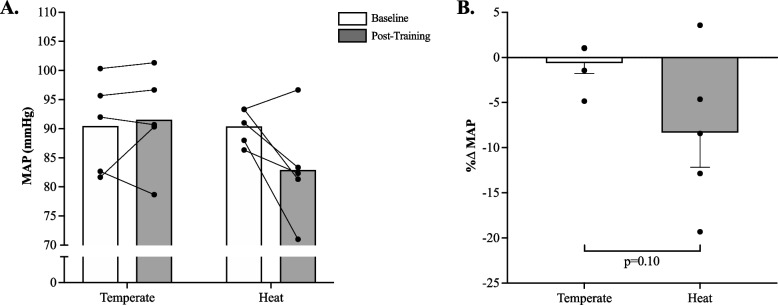
**A** Mean Arterial Pressure at baseline and post-HIIE training with or without heat acclimation (Cohen’s d = 1.2); **B** percent change in MAP (Cohen’s d = 1.5). HIIE-T (*n* = 5; females = 3, males = 2) and HIIE-H (*n* = 5; females = 3, males = 2). A one-tailed Mann–Whitney U Test was used to identify any potential group differences in relative changes from baseline (panel **B**). Data presented mean ± SEM

There was no significant (*p* = 0.07, η^2^ = 0.08) temperature x time interaction for peripheral MAP (Fig. 4A), although there appeared to be a trend towards reduction in MAP in the HIIE-H group, only. However, there was no significant main effect of temperature (*p* = 0.35, η^2^ = 0.08) or time for peripheral (*p* = 0.15, η^2^ = 0.04) MAP. When expressed as percent change from baseline, non-parametric t-tests did reveal a non-significant reduction in pMAP in HIIE-H group only (Fig. 4B; *p* = 0.10, Cohen’s d = 1.5).

### Arterial stiffness

**Fig. 5:**
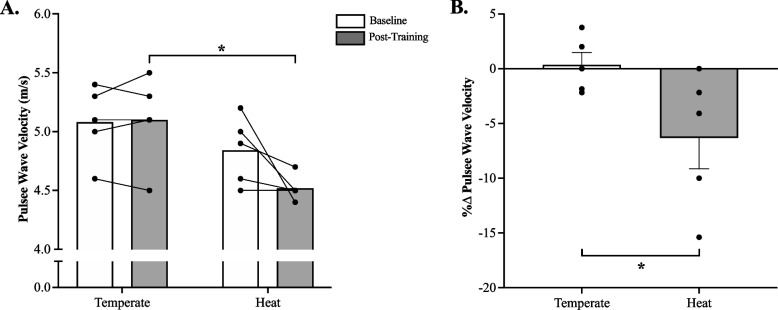
**A** Pulse Wave velocity (PWV) at baseline and post-HIIE training with or without heat acclimation (Cohen’s d = 1.6); **B** percent change in PWV (Cohen’s d = 1.5). HIIE-T (*n* = 5; females = 3, males = 2) and HIIE-H (*n* = 5; females = 3, males = 2). *p < 0.05. A one-tailed Mann–Whitney U Test was used to identify group differences in relative changes from baseline (panel **B**). Repeated measures 2 × 2 ANOVA was used to identify any potential main effects and interactions (panel **A**). Data presented mean ± SEM

There was no significant (*p* = 0.06, η^2^ = 0.06) temperature x time interaction for PWV (Fig. 5). However, a significant main effect of temperature was detected. Although there was no significant (*p* > 0.05) between-group difference for PWV at baseline, a significantly lower PWV was documented in the HIIE-H group post-training as compared to the HIIE-T group (Fig. 5A; *p* = 0.04, η^2^ = 0.35, Cohen’s d = 2.4). There was no significant main effect of time for PWV (*p* = 0.09, η^2^ = 0.05). When expressed as a percent change from baseline, non-parametric t-tests did reveal a significant reduction in PWV in the HIIE-H group (Fig. 5B; *p* = 0.03, Cohen’s d = 1.6).

### Performance: VO_2peak_ & 5 km time-trial

**Fig. 6:**
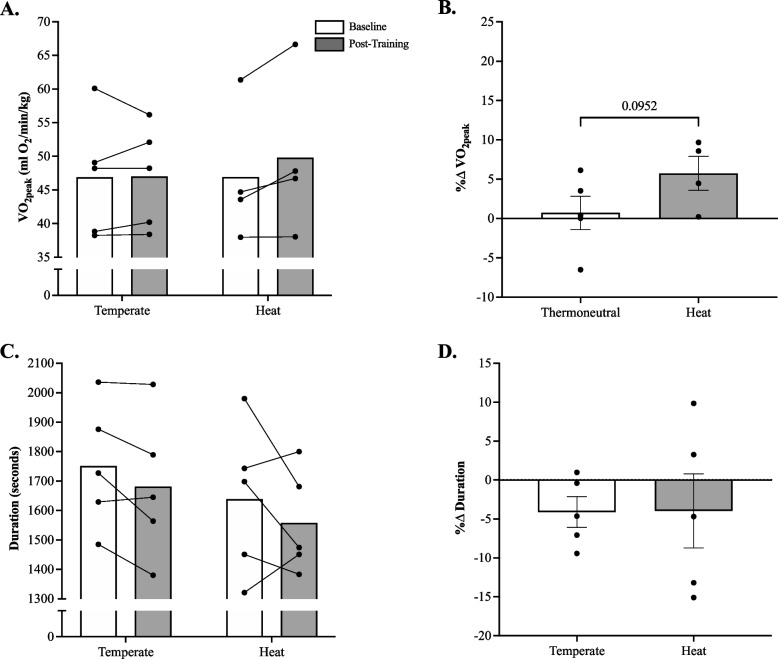
**A** Peak oxygen consumption (VO_2peak_; Cohen’s d = 0.3) and **C** 5 km time-trial duration (Cohen’s d = 0.4) at baseline and post-HIIE training with or without heat acclimation; **B** percent change in VO_2peak_ (Cohen’s d = 1.2) and (**D**) 5 km time-trial duration (Cohen’s d = 0.1). The sample size for the heat group is *n* = 4 for VO_2peak_ absolute and % change in panels A. and B. When the data were pooled (HIIE-T + HIIE-H), there was a trend towards a reduction in 5 km time-trial performance with HIIE training (Pre: 1695 ± 231 vs. Post: 1620 ± 210 s; Cohen’s d = 0.40, *p* = 0.0511). HIIE-T (*n* = 5; females = 3, males = 2) and HIIE-H (*n* = 5; females = 3, males = 2). A one-tailed Mann–Whitney U Test was used to identify group differences in relative changes from baseline (panel **B** and **D**). Data presented mean ± SEM

There was no significant (*p* = 0.90, η^2^ < 0.001) temperature x time interaction for 5 km time-trial performance (Fig. 6C). Also, there was no significant main effect of temperature (*p* = 0.41, η^2^ = 0.07) or time (*p* = 0.12, η^2^ = 0.03) for 5 km time-trial performance. Likewise, no significant difference in 5 km time-trial performance was documented when expressed as a percent change from baseline (*p* = 0.49, Cohen’s d = 0.02; Fig. 5D). However, when the data were pooled (HIIE-T + HIIE-H), there was a trend towards a 4% reduction in 5 km time-trial performance with HIIE training (Pre: 1695 ± 231 vs. Post: 1620 ± 210 s; Cohen’s d = 0.40, *p* = 0.051). This pooled analysis was conducted to ensure that the present training stimulus (i.e., HIIE) was sufficient to elicit training adaptations as has been extensively reported in the literature [48, 49]. RPE and TS data pre-, post-training, and during each training session, were unremarkable in terms of training and/or group effect (data not shown).

Data on only 4 participants in the HIIE-H group were included in the final analysis for VO_2peak_ given potential equipment error for one participant. There was no significant (*p* = 0.08, η^2^ = 0.02) temperature x time interaction for VO_2peak_ (Fig. 6A). Also, there was no significant main effect of temperature or time (*p* = 0.06, η^2^ = 0.02) for VO_2peak_. Furthermore, the percent change from baseline VO_2peak_ was not significantly different between groups according to the non-parametric t-test (Fig. 6B; *p* = 0.10, Cohen’s d = 1.4).

## Discussion

The present pilot study examined, in active young, healthy adults, the potential interaction of low-volume HIIE and short-term heat stress on the autonomic nervous system, markers of CV function, and aerobic endurance performance. The main findings of this study are that six sessions of HIIE with added heat exposure: 1) elicited significant reductions in arterial stiffness, as measured by pulse wave velocity, that were of large effect size, 2) markedly decreased central and peripheral systolic BP (~ 7–8 mmHg decrease in MAP), which, in the absence of a change in resting HR and HRV, suggests a decrease in total peripheral resistance, but 3) did not significantly improve aerobic function, assessed as VO_2peak_ and 5-km treadmill time trial performance, as compared to an intensity-matched temperate HIIE group. Together, these data demonstrate a potential added benefit of heat exposure to high-intensity exercise training on CV health. This exercise paradigm is therefore an enticing training strategy that warrants further investigation.

### HIIT & short-term heat stress: aerobic performance

In non-acclimated athletes, the thermoregulatory imposed by heat stress, in both hot and temperate conditions, is pronounced and a known deterrent to exercise performance [50]. This has roused interest in different training modalities to sustain HA and exercise performance, and in particular, the application of combined high-intensity exercise and heat to amplify training adaptations. As little as five, 27-min sessions of cycle ergometry HIIT at temperatures of 22 °C, 36% RH was sufficient to improve RPE and TC during a submaximal exercise test in the heat in elite Australian football athletes (VO_peak_: ~ 48 ml/kg/min) [23]. However, resting HR, T_core_, and VO_2peak_ were unaffected, suggesting only partial HA. Likewise, 3 weeks of high-intensity training 3 sessions per week (~ 33˚C) improved 3 km running performance in temperate condition outdoor by 3.3% [31]. However, this beneficial effect on performance was delayed, reaching significance 3 weeks after the intervention, i.e., when hematological adaptations had weaned off, such that no clear underlying physiological mechanisms could be identified. Thermoperceptual adaptations in the short-term (≤ 5 days) have been documented in elite cyclists [28] (VO_2peak_: > 55 ml/kg/min) and active young adults (VO_2peak_: ~ 3.75 l/min) [16], with reductions in resting HR and rectal temperature suggesting rapid and full HA in both studies. While these adaptations undoubtedly result in improved performance in hot conditions [7, 10, 12–14], it is unclear if they provide additional benefits in temperate conditions. Interestingly, failure to observe improvements in indices of aerobic performance (e.g., Cooper Test) [15], and even an impaired cycling capacity due to overreaching in the study by Reeve et al. calls into question the performance efficacy of this HA paradigm. In addition, using a parallel study design, Karlsen et al. reported no effects of 2 weeks of HA (34˚C) on VO_2max_ and 43 km cycling performance test in temperate conditions outdoor in competitive cyclist training ~ 15 h. a week, including 2.5 h. of high-intensity exercise [30].

The disparity between thermophysiological and performance adaptions is in agreement with the present results. Resting HR, as well as RPE and TS, were unaffected by the current HIIE-H protocol. Although thermophysiological alterations respond positively to as little as 5 days of thermal exposure [51], the present data suggests our thermal stimuli (6 days of low-volume HIIE-H) was inadequate to induce full HA. Nevertheless, improvements in BP, MAP, and PWV underscore that, irrespective of the degree of HA, HIIE-H induced favorable modifications in CV function. This translated to a nonsignificant 6% increase in VO_2peak_, which matches the 5–8% increase in VO_2max_ documented by Lorenzo et al. using a longer HA in trained cyclists, and opposes Kelly et al., the latter using a similar HIIT protocol to the present study in profession football athletes. Importantly, however, is that the change in VO_2peak_ was nonsignificant and likewise did not reflect in an improvement in 5 km time-trial performance. Wardenaar et al. also reported an insignificant, yet meaningful, 4% improvement in Cooper Test performance with a 5-day HA program in collegiate athletes, and it has been argued that longer duration exercise stimuli (10 vs. 5 days) is needed for aerobic performance adaptations to ensue [40]. Such differences in exercise-heat intensity and duration likely explain the incongruent results, which warrants further research.

### HIIT & heat stress: blood pressure

Although HR was unaffected by HIIE with heat stress, the improved BP profile suggests a cardioprotective benefit to this exercise-heat paradigm, albeit only in a small group of individuals. However, this reduction in BP parallels that observed with long-term PHT protocols, which have demonstrated improvements in vascular function and BP regulation in various populations [52]. For example, Brunt et al. demonstrated a -10-mmHg improvement in MAP, owing to decreased arterial stiffness and improved endothelial function, with eight weeks of passive heat therapy in young sedentary adults [20]. Likewise, MAP tended to improve at a similar magnitude (-8 mmHg) after the HIIT-H intervention and was accompanied by a reduction in PWV (-0.3 m/s), a well-established marker of vascular stiffness. Although nonsignificant, the ~ 6% improvement in MAP does hold clinical value, and more importantly, follows the -7 and -10 mmHg reductions in resting central and peripheral SBP, respectively. Thus, it appears that six sessions of HIIE under short-term thermal load may improve BP profile, similar to the effects of several weeks (40 session, 90 min/session) of acute whole-body heat therapy [19] in healthy, middle-aged adults, but warrants further investigation which a larger cohort of participants.

To the best of our knowledge, this is the first study to investigate BP and, indirectly, vascular alterations to HIIE under heat stress. Previous exercise HA studies have reported improvements in CO, and consequently, aerobic exercise performance (VO_2max_), likely attributed to PV expansion [11, 14]. PV expansion, in theory, could increase BP and thus CV event risk. Nevertheless, the reduction in BP and MAP in the present study, with no change in HRV (e.g., no change in cardiac autonomic function), suggests a capacity of the systemic circulation to enhance cardiac contractile function in the face of PV expansion. Therefore, a potential for a decrease in peripheral resistance induced by combined HIIE and heat stress appear outweigh the potential deleterious effects of increased PV on BP regulation. Future research assessing both PV and BP are needed as we are limited by a lack of PV data in the current study. However, such improvements in BP may be of clinical importance, especially when considering factors such as adherence, thermal tolerance, and the ability to safely induce CV adaptions in a time-efficient manner, which warrants further attention.

### HIIT & heat stress: cardiac autonomic function

HRV is a non-invasive assessment of cardiac autonomic balance (i.e., sympathetic and parasympathetic tone) [43], providing a unique window into autonomic nervous system factors regulating adaptations to physiological insults (e.g., heat). The marginal and nonsignificant increase in HRV, coupled with no change in HR, in the HIIE-H group therefore suggests little or no role of neural adaptions, specifically estimated cardiac autonomic function. This contrasts with the 10% increase in HRV, and 5% reduction in HR, that has been observed with 11 days of on-field soccer training in hot humid conditions in well-trained male soccer athletes [53]. In conjunction with PV expansion and an enhanced CO [11], previous studies suggest that mitigation of sympathetic neural drive to cardiac muscle is one physiological means by which myocardial efficiency is enhanced with exercise in the heat (Frank-Starling Mechanism: CO = SV X HR). This disparity may allude to different mechanisms of adaptions for the different heat, duration (long- vs. short-term) and exercise (high- vs. moderate-intensity) modalities. Indeed, follow-up studies that directly measure PV, CO, and HRV following low-volume HIIT with heat are required to test this hypothesis.

### HIIT & heat stress: arterial stiffness

In line with the reduced peripheral resistance hypothesis, there was a significant 6% decrement in PWV in our heat group. PWV is a validated assessment of arterial stiffness, the latter an independent risk factor for CV diseases (e.g., atherosclerosis) [54], and the former itself a strong predictor of CV morbidity and mortality. In accordance with our findings, eight weeks of Hot Yoga (40.5 °C, 40–60% RH) reduced brachial-ankle PWV to a similar magnitude (~ 0.5 m/s), albeit in older [55] and overweight/obese adults [56]. Thus, although our practical (but nonsignificant) ~ 0.3 m/s improvement in PWV was short of the 1 m/s observed with 12 weeks of whole-body hot water immersion and deemed clinically significant [19], this response was elicited in a markedly shorter timeframe and in healthy individuals. Collectively, in the present HIIE-H paradigm, factors downstream from central neural drive appear to be the dominant mechanism at play, and specifically, suggests enhanced endothelial function, perhaps mediated through heat shock proteins (HSP) [57] and transient receptor potential cation channel subfamily V (TRPV) channel [58] augmenting endothelial nitric oxide synthase activity and NO production. This hypothesis will benefit from future studies that interrogate peripheral vascular resistance with robust assessments of vascular endothelial function, such as flow-mediated dilation (FMD) and passive leg movement (PLM) and assessment of circulating factors (e.g., HSPs) that might be explanatory in the enhanced vascular endothelial function, that may be superior with HIIE-H.

#### Experimental considerations

Several methodological considerations might explain the disparity in the present results. The small sample size (*n* = 10) may not be statistically powered to detect performance adaptations, although several physiological variables were significantly altered (VO_2peak_, PWV, pSBP, cSBP) and establish effect sizes for sample size estimations in subsequent studies. Importantly, the application of continuous (90 min), low-moderate intensity (50% VO_2max_) exercise, at a higher frequency (≥ 10 days), might allow for a stronger training and heat stimulus. It is therefore conceivable that our lower volume HIIE protocol was appropriate to improve aerobic capacity and CV function at almost half the frequency (6 vs. 10 days) of traditional LTHA protocols, but not sufficient to induce performance adaptations. Future studies should explore longer interventions of HIIT with heat stress to test this hypothesis. Furthermore, it has been shown that females exhibit slower HA kinetics (e.g., increased active sweat gland concentration) and performance adaptations (e.g., improved power output) than males [59]. We were underpowered to investigate sex differences and to account for sex as a covariate, but it is possible that there may be sex specificity (e.g., time-trial performance) and certain trends (e.g., MAP) driven by female participants (*n* = 6). This is a topic that has recently garnered traction in the heat literature, warranting further attention. Lastly, a better understanding of the role of dietary and physical activity habits, as well as familiarization and reproducibility of the tests performed, is needed to contextualize the effects of heat and HIIE.

## Conclusions

Despite no appreciable differences in HR and indices of HRV (i.e., SDNN and RMSSD), six sessions of HIIE in hot-humid conditions significantly improved central and peripheral BP, and accordingly, MAP, compared to HIIE performed at the same relative intensity in temperate conditions. These effects appeared to be mediated by decreased arterial stiffness, as indicated by reductions in PWV, and, perhaps, an enhanced vascular endothelial function. Importantly, contrary to traditional exercise-heat paradigms of long duration (> 60 min) and frequency (> 36 sessions), the utilization of low-volume HIIT elicited favorable CV and performance adaptations of similar magnitude in a time-efficient manner. Furthermore, given that adaptations were documented in young, healthy, and recreationally active participants, there is a potential for this exercise-heat paradigm with clinical populations in need of a rapid and potent intervention to improve their CV risks profile, proposing an enticing avenue for future research with careful safety monitoring.

## Data Availability

Upon reasonable request, the corresponding author may grant access to the raw data.

## Abbreviations

HIIE: High intensity interval exercise
VO2peak: Peak oxygen consumption
HIIE-H: High intensity interval exercise in the heat
HIIE-T: High intensity interval exercise in temperate conditions
RH: Relative humidity
HR: Heart rate
HRV: Heart Rate Variability
BP: Blood pressure
pBP: Peripheral blood pressure
cBP: Central blood pressure
pMAP: Peripheral mean arterial pressure
PWV: Pulse wave velocity
cSBP: Central systolic blood pressure
pSBP: Peripheral systolic blood pressure
CV: Cardiovascular
HA: Heat acclimation
TCore: Core temperature
VO2max: Maximal oxygen consumption
PV: Plasma volume
SV: Stroke volume
CO: Cardiac output
LTHA: Long-term Heat Acclimation
HIIT: High intensity interval training
USG: Urine specific gravity
RPE: Rating of perceived exertion
TS: Thermal sensation
RMSSD: Root mean square of successive differences
SDNN: Standard deviation of n–n intervals
